# A clinical model for identifying the short-term risk of breast cancer

**DOI:** 10.1186/s13058-017-0820-y

**Published:** 2017-03-14

**Authors:** Mikael Eriksson, Kamila Czene, Yudi Pawitan, Karin Leifland, Hatef Darabi, Per Hall

**Affiliations:** 1 Department of Medical Epidemiology and Biostatistics, Karolinska Institutet, Box 281, Stockholm, 171 77 Sweden; 2 Department of Radiology, South General Hospital, 118 83 Stockholm, Sweden; 3 Department of Oncology, South General Hospital, 118 83 Stockholm, Sweden

**Keywords:** Risk prediction, Breast cancer, Mammographic density, Microcalcification, Masses, Computer-aided detection, Prevention

## Abstract

**Background:**

Most mammography screening programs are not individualized. To efficiently screen for breast cancer, the individual risk of the disease should be determined. We describe a model that could be used at most mammography screening units without adding substantial cost.

**Methods:**

The study was based on the Karma cohort, which included 70,877 participants. Mammograms were collected up to 3 years following the baseline mammogram. A prediction protocol was developed using mammographic density, computer-aided detection of microcalcifications and masses, use of hormone replacement therapy (HRT), family history of breast cancer, menopausal status, age, and body mass index. Relative risks were calculated using conditional logistic regression. Absolute risks were calculated using the iCARE protocol.

**Results:**

Comparing women at highest and lowest mammographic density yielded a fivefold higher risk of breast cancer for women at highest density. When adding microcalcifications and masses to the model, high-risk women had a nearly ninefold higher risk of breast cancer than those at lowest risk. In the full model, taking HRT use, family history of breast cancer, and menopausal status into consideration, the AUC reached 0.71.

**Conclusions:**

Measures of mammographic features and information on HRT use, family history of breast cancer, and menopausal status enabled early identification of women within the mammography screening program at such a high risk of breast cancer that additional examinations are warranted. In contrast, women at low risk could probably be screened less intensively.

**Electronic supplementary material:**

The online version of this article (doi:10.1186/s13058-017-0820-y) contains supplementary material, which is available to authorized users.

## Background

Risk prediction models for breast cancer use lifestyle factors [1], family history of breast cancer [2], mammographic density [3], genetic determinants [4], or any combination of these factors to predict risk of developing the disease [3]. Mammographic density is one of the strongest risk factors for breast cancer and consists of the radiographically dense fibroglandular part of the mammogram. Women with dense breasts have both an increased risk of breast cancer and a lesser likelihood of a cancer being detected. It is currently mandatory by law to report the level of mammographic density to a woman undergoing a mammography in 27 U.S. states, but there is no obligation to report the risk of breast cancer.

Computer-aided detection (CAD) is designed to support radiologists at mammographic screening units in diagnosing early breast cancer. These software can indicate suspicious microcalcifications and masses. We used fully automated CAD and breast density measurement systems and predicted the probability for a woman with a negative mammogram result to be diagnosed with breast cancer within 2 years. We wanted to create an easily implementable prediction tool for individualized breast cancer screening without adding substantial cost or effort to the health care system.

We merged established risk factors, such as use of hormone replacement therapy (HRT), family history of breast cancer, menopausal status, body mass index (BMI), and mammographic density with microcalcifications and masses, using U.S. Food and Drug Administration-approved CAD software [5]. We were able to identify high-risk women who would probably benefit from intensified breast cancer screening or would be in immediate need of clinical examinations. In parallel, we identified women with such a low breast cancer risk that they might not benefit from screening. To achieve these goals, we used a unique, prospective Swedish population-based screening cohort: the Karolinska Mammography Project for Risk Prediction of Breast Cancer (KARMA) cohort (karmastudy.org).

## Methods

In Sweden, women aged 40–74 years are invited every 18–24 months to the national screening program [6]. Women who attended mammographic screening at four hospitals in Sweden were invited to be included in the KARMA cohort between January 2011 and March 2013. A total of 70,877 women chose to participate (age range 31–79 years) [7]. Participants answered a comprehensive web-based questionnaire, donated blood, allowed storage of mammograms, and accepted linkage to national breast cancer registers. By October 2015, a total of 570 incident breast cancers had been identified. Women diagnosed with breast cancer within 3 months of a negative entry mammogram were omitted because it could not be excluded that a cancer was detected at the screening visit. A total of 137 patients lacked information on one or several risk factors, leaving 433 breast cancer cases to be used for the model development. However, the 137 women lacking information were included in calculating the absolute risk estimates, whereby missing data were replaced with the average risk of that risk factor. Four control subjects were matched on age to each case in a prospective nested case-control design.

Full-field digital mammograms from the mediolateral oblique (MLO) and craniocaudal (CC) views of the left and right breasts were used to measure mammographic density using the area-based STRATUS method (Additional file 1: Supplementary methods 1). The percentage mammographic density was calculated by dividing the dense area by the total breast area. Breast density was categorized on scale cutpoints (2%, 18%, 49%) into four breast composition groups reflecting the clinically accepted Breast Imaging Reporting and Data System (BI-RADS; American College of Radiology, Reston, VA, USA) score [5, 8–10] (Additional file 1: Supplementary methods 2). The computer-generated score is hereafter called *cBIRADS*.

The CAD software (M-Vu CAD®; iCAD, Nashua, NH, USA) identifies suspicious microcalcifications and masses and presents the findings to the radiologist or as digital text information. Raw mammograms of the MLO and CC views of right and left breasts were used to identify microcalcifications and masses. On the basis of the distribution of microcalcifications among control subjects, the number of microcalcifications was categorized into five groups: 0, 1–10, 11–20, 21–40, and >40 microcalcifications. The number of masses was given as the true number. Level of density and number of microcalcifications and masses, as well as the differences in density and number of microcalcifications and masses between breasts, were used in the model.

On the basis of self-reported information, dichotomous variables were created for current use of HRT, history of breast cancer in first-degree relatives, and menopausal status. Current use of HRT was defined as use within the last 12 months. BMI and age were assessed at the time of study entry, which was the time the baseline mammogram was taken. Screening-detected breast cancer was defined as breast cancer diagnosed within 3 months of a screen. An interval breast cancer was defined as a breast cancer diagnosed at least 3 months after a negative screen but before the date of the next scheduled screen [11].

Descriptive statistics were presented for participant characteristics and to describe mammographic features in the tumor breast side (where the tumor eventually was diagnosed) versus the nontumor breast side in the cases. Differences between the breasts were calculated without assuming knowledge of the tumor breast side. These absolute differences were calculated as the standard deviation (SD) of the two breasts for each mammographic feature and were used as continuous predictors in the final model.

The continuous predictors in the conditional logistic regression model were tested for the best transformation using the Sauerbrei method [12] with fractional polynomials, and the predictors for the absolute breast differences were transformed as reciprocal numbers. The functional form of the final model was assessed using the branch-and-bound Furnival and Wilson statistics for main effects and interaction terms [13]. Relative risks were reported as HRs in this prospective study design.

Absolute risks were calculated using the Individualized Coherent Absolute Risk Estimator (iCARE) package in R [14]. The Swedish national incidence rates of breast cancer and competing mortality risks were used and calculated as the average rates from 2007 to 2011. Prevalence rates of HRT use and family history of breast cancer were derived from the KARMA cohort, and the relative risks from the regression analyses were entered into the model matrix. Missing data from nonreported risk factors were imputed with model averaged risk estimates using the iCARE protocol (Additional file 1: Supplementary methods 3).

Using the same data, a cross-validated AUC was calculated and compared with values generated by the established Tyrer-Cuzick and Gail risk models. The numbers of invasive and in situ cases that were diagnosed during follow-up were tabulated by quintile of the 2-year absolute risks predicted at baseline. The increase in number of diagnosed cases by quintile of baseline risk was calculated and tested for linear trend.

All statistical tests were two-sided at a significance level of 0.05 and calculated using SAS version 9.4 software (SAS Institute, Cary, NC, USA) for descriptive statistics and relative risks. Absolute risks were evaluated with R 3.3.0 software using the iCARE package 1.0.0.

## Results

In all, 433 women had a negative mammogram result more than 3 months prior to diagnosis and had full information on risk factors. The data of these women were used to develop the model (Table 1). The median follow-up time between the baseline mammogram and diagnosis of breast cancer was 1.7 years, mean age at breast cancer diagnosis was 59.0 years, 88% of the breast cancers were invasive, and 63% were detected by screening. Significantly more cases were current users of HRT (6.9% in cases and 4.4% in control subjects, *p* = 0.05) and had a family history of breast cancer (19% of cases and 13% of control subjects, *p* = 4.5 × 10^−4^) (Table 1).

**Table 1:** Characteristics of cases and control subjects

Study participant characteristics	Cases	Control subjects	*p* Value^a^
Number of women	433	1732	–
Age at breast cancer diagnosis, mean (SD)	59.0 (9.4)	–	–
Years from mammography to breast cancer, median	1.74	–	–
Invasive breast cancer, %	88	–	–
Screening detected breast cancer, %	63	–	–
Age at mammography, mean (SD)	57.4 (9.2)	57.4 (9.2)	0.99
BMI, mean (SD)	25.6 (4.6)	25.3 (4.0)	0.19
Age at menarche, mean (SD)	13.1 (1.4)	13.2 (1.5)	0.61
Parity, %	89	88	0.56
Age at first birth, mean (SD)	27.1 (5.4)	26.6 (5.2)	0.11
Current use of HRT, %	6.9	4.4	0.05
Postmenopausal, %	65	65	0.78
Breast cancer in family, %	19	13	4.5 × 10^−4^

*BMI* Body mass index, *HRT* Hormone replacement therapy

^a^ *p* Values for means were calculated with Student’s *t* test, medians with Wilcoxon rank-sum test, and percentages with the chi-square test

At baseline, the median mammographic density was 23.0% in cases on the tumor side (i.e., on the side where the tumor was diagnosed at follow-up) and 12.2% in control subjects (*p* = 4.0 × 10^−10^) in the breast corresponding to the tumor side in cases (Table 2). The corresponding figures for the contralateral side in cases and control subjects were 21.7% and 12.5%, respectively (*p* = 2.5 × 10^−7^). Comparing density pairwise between the tumor side and nontumor side in cases showed a mean difference of 1.1% (*p* = 3.4 × 10^−3^) (Table 2).

**Table 2:** Mammographic features in tumor and nontumor side in cases and control subjects

Mammographic features	Cases (*n* = 433)	Control subjects (*n* = 1732)	*p* Value^a^
Percentage mammographic density on tumor side, median (IQR)	23.0 (6.1–44.1)	12.2 (2.4–32.8)	4.0 × 10^−10^
Percentage mammographic density on nontumor side, median (IQR)	21.7 (5.1–43.4)	12.5 (2.7–33.2)	2.5 × 10^−7^
Tumor vs. nontumor side, percentage mammographic density	1.1 (7.8)	–	3.4 × 10^−3^
Number of microcalcifications on tumor side, mean (SD)	6.1 (15.3)	2.6 (13.1)	4.0 × 10^−20^
Number of microcalcifications on nontumor side, mean (SD)	3.4 (13.0)	2.6 (12.2)	0.03
Tumor vs. nontumor side, microcalcifications	2.7 (17.9)	–	1.9 × 10^−3^
Number of masses on tumor side, mean (SD)	0.77 (0.92)	0.56 (0.76)	8.4 × 10^−6^
Number of masses on nontumor side, mean (SD)	0.51 (0.75)	0.55 (0.78)	0.39
Tumor vs. nontumor side, masses	0.26 (1.1)	–	9.2 × 10^−3^
Individual absolute difference between breasts^b^			
Percentage mammographic density, mean (SD)	3.8 (4.0)	3.1 (3.7)	1.7 × 10^−6^
Microcalcifications, mean (SD)	2.9 (6.1)	1.6 (5.7)	4.0 × 10^−16^
Number of masses, mean (SD)	0.33 (0.42)	0.28 (0.40)	0.02

^a^ *p* Values of median values were calculated with Wilcoxon rank-sum test. *p* Values of means were calculated with Student’s *t* test. Mediolateral oblique and craniocaudal view mammograms are used. The individual microcalcifications are within calcification cluster(s)

^b^Absolute difference between the two breasts was calculated as the standard deviation SD of density of the left and right breasts for each woman

The mean number of microcalcifications in cases and control subjects was significantly different on both the tumor side (for cases versus corresponding side for control subjects, 6.1 vs. 2.6; *p* = 4.0 × 10^−20^) and the contralateral side in cases and control subjects (3.4 vs. 2.6, *p* = 0.03). The comparison between tumor and nontumor sides in cases showed a mean difference of 2.7 microcalcifications (*p* = 1.9 × 10^−3^) (Table 2).

The mean number of detected masses in cases versus control subjects was significantly different on the tumor side (for cases and corresponding side for control subjects, 0.77 vs. 0.56; *p* = 8.4 × 10^−6^) but not on the contralateral side in cases and control subjects. The pairwise comparison between tumor and nontumor sides in cases showed a mean difference of 0.26 masses (*p* = 9.2 × 10^−3^) (Table 2).

In the lower part of Table 2, the absolute differences between the breasts are presented to contrast cases and control subjects. It can be seen that cases have a more uneven distribution of mammographic density (*p* = 1.7 × 10^−6^), microcalcifications (*p* = 4.0 × 10^−16^), and masses (*p* = 0.02).

Relative risks of breast cancer within 3 years from a negative mammographic screening result at baseline were calculated using two models (Table 3). In the fully adjusted model, the risk of breast cancer in women with a family history of the disease was 1.3 (95% CI 1.0–1.7). A significant difference was seen for women with the highest versus lowest cBIRADS scores (HR 4.8), in women with microcalcifications in category 4 compared with no microcalcifications (HR 2.0), in women with significant difference in density (HR 1.9), and in microcalcifications (HR 2.8) between left and right breasts (Table 3). A more detailed stratification is provided in Additional file 1: Table S1.

**Table 3:** Relative risks of breast cancer within 3 years of a negative mammographic screening result in relation to use of hormone replacement therapy, family history of breast cancer, and mammographic features

Study participant and mammographic features	HR^a^ (95% CI)	HR^b^ (95% CI)
Current use of HRT (same-year user vs. previous or nonuser)	1.4 (0.9–2.1)	1.3 (0.9–2.0)
Family history of breast cancer	1.3 (1.1–1.7)	1.3 (1.0–1.7)
Percentage mammographic density (cBIRADS 4 vs. 1)	4.9 (2.8–8.6)	4.8 (2.6–8.8)
Percentage mammographic density (per SD)	1.6 (1.4–1.8)	1.6 (1.4–1.8)
Number of microcalcifications^c^ (category 4 vs. 0)	2.0 (1.3–3.1)	2.0 (1.3–3.2)
Number of masses (4 vs. 0)	1.7 (0.8–3.5)	1.7 (0.8–3.5)
Individual absolute difference between breasts^d^		
Percentage mammographic density	3.4 (2.2–5.2)	1.9 (1.2–3.0)
Number of microcalcifications	2.5 (1.9–3.1)	2.8 (1.8–4.5)
Number of masses	1.4 (0.9–2.2)	1.1 (0.6–1.9)

*HRT* Hormone replacement therapy

^a^Adjusted for age, body mass index

^b^Adjusted for age, body mass index, mammographic density, microcalcifications, masses, breast cancer in family, menopausal status, and current use of HRT

^c^Category 0 means 0 microcalcifications, and 1 is 1–10 microcalcifications. The corresponding numbers for 2, 3, and 4 are 11–20, 21–40, and >40 microcalcifications, respectively

^d^Absolute difference between right and left breasts was calculated as the standard deviation SD of the breasts for each mammographic feature

Dividing cases into invasive (*n* = 383) and in situ (*n* = 50) cancers (Additional file 1: Table S2) revealed that microcalcifications were significantly more likely to identify risk of future cancer in situ than invasive cancers (*p* = 0.03 for number of microcalcifications and *p* = 0.01 for absolute difference in microcalcifications between breast sides). When stratifying on mode of detection (i.e., screening-detected [*n* = 275] vs. interval [*n* = 158] breast cancers), we observed that all mammographic features, including the absolute differences between the breasts, were more likely to identify interval cancers than screening-detected cancers (Additional file 1: Table S2). Women with a cBIRADS score of 4, microcalcifications in category 3 or higher, and three or more masses had a nearly ninefold higher risk of breast cancer than women with a cBIRADS score of 1 and no microcalcifications or masses (Table 4).

**Table 4:** Relative risk of developing breast cancer in relation to the combined effect of mammographic density, number of microcalcifications, and number of masses

Mammographic features combined	HR^a^ (95% CI)	HR^b^ (95% CI)
1. cBIRADS 1, microcalcification category 0^c^, 0 masses, reference	1.0	1.0
2. cBIRADS 2, microcalcification category 1, 1 masses	4.2 (2.5–7.1)	4.3 (2.4–7.5)
3. cBIRADS 3, microcalcification category 2, 2 masses	7.9 (4.3–14.4)	7.9 (4.2–15.2)
4. cBIRADS 4, microcalcification category ≥3, ≥3 masses	8.0 (4.5–14.3)	8.7 (4.7–16.0)

*cBIRADS* Computer-generated Breast Imaging Reporting and Data System score

^a^Adjusted for age, body mass index

^b^Adjusted for age, body mass index, family history of breast cancer, menopausal status, and current use of hormone replacement therapy

^c^Category 0 means 0 microcalcifications, and 1 is 1–10 microcalcifications. The corresponding numbers for 2, 3, and 4 are 11–20, 21–40, and >40 microcalcifications, respectively

**Fig. 1:**
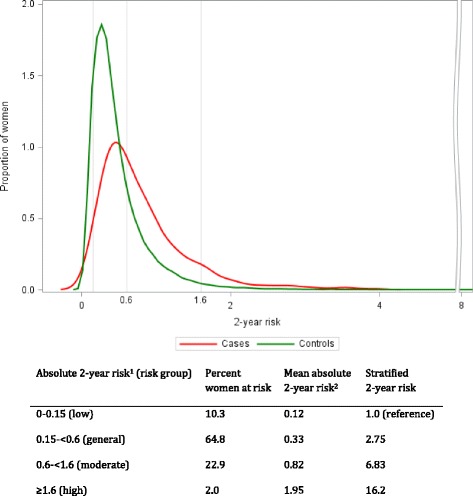
Frequency distribution of 2-year absolute risks for developing breast cancer in cases and control subjects in the KARMA cohort. ^1^cut-offs for the general, moderate, and high-risk groups are based on the NICE guidelines for 10-year risk in age group 40 - 50 (<3%, 3-8%, >8%) divided by 5. We added a fourth low risk group with the absolute risk cut-off 0.15. ^2^calculation based on relative risks from the case – control dataset, the KARMA cohort prevalence of risk factors and competing risks

The final model including the selected risk factors, stratified by menopausal status, is provided in Additional file 1: Table S3 and was used for calculating absolute risks. We plotted the frequency distribution of the predicted absolute risk of breast cancer using the generated relative risks and prevalence of risk factors in 570 incident breast cancer cases and 60,237 healthy women in the KARMA cohort (Fig. 1). This was done after exclusion of women with previous breast cancers and/or lack of mammograms (Additional file 1: Supplementary methods 3).

To conform to the National Institute for Health and Care Excellence guidelines [15], we divided the 10-year risk cutoffs (general, moderate, high) by 5 to get 2-year risk cutoffs (i.e., <0.6%, 0.6% to <1.6%, and ≥1.6%). Because the <0.6% risk group included 75% of the women, the group was further divided into a low-risk group (<0.15% 2-year risk). The mean absolute 2-year risks of breast cancer in the different risk categories were 0.12%, 0.33%, 0.82%, and 1.95%, equivalent to a 16-fold difference comparing the highest- with the lowest-risk groups (Fig. 1).

The AUCs measured were 0.63 (95% CI 0.60–0.65) using mammographic density adjusted for BMI and age at mammography, 0.64 (95% CI 0.62–0.67) when adding family history of breast cancer and HRT use, and 0.71 (95% CI 0.69–0.73) after adding microcalcifications and masses (Table 5).

**Table 5:** Discrimination performance of final model and sub models in comparison to established risk models

Model	AUC^a^	95% CI	LR^b^
1. Percentage mammographic density, age at mammography, BMI	0.63	0.60–0.65	109.2
2. Model 1 + family history of breast cancer, HRT use	0.64	0.62–0.67	122.0
3. Model 2 + absolute differences for calcifications, masses, density	0.70	0.68–0.72	193.7
4. Model 3 + interaction between percentage density and masses	0.71	0.69–0.73	233.6
Established risk models for comparison			
Tyrer-Cuzick^c^	0.63	0.60–0.65	88.2
Gail^d^	0.56	0.53–0.58	34.9

*BMI* Body mass index, *HRT* Hormone replacement therapy, *LR* Likelihood ratio

^a^AUC was evaluated for the absolute risks of stated models

^b^Chi-square test of β = 0

^c^Tyrer-Cuzick model included risk factors of age, age at menarche, age at first child, menopause, length, weight, HRT, hyperplasia, atypical hyperplasia, lobular cancer in situ, and first-/second-degree family history of breast cancer. Data coding was done according to the Tyrer-Cuzick protocol

^d^Gail model included risk factors of age, age at menarche, age at first live birth, number of previous breast biopsies, atypical hyperplasia, and first-degree family history of breast cancer. Data coding was done according to the Gail protocol

There was a significant linear trend in the association between increasing 2-year absolute baseline risk and larger proportion of cancers diagnosed during the study follow-up. For each quintile of 2-year baseline risk, 56.7 more cases were found to be diagnosed (*p* = 0.04). The corresponding numbers for the Tyrer-Cuzick and Gail models were 35.3 cases (*p* = 0.01) and 15.1 cases (*p* = 0.14), respectively (Additional file 1: Table S4).

## Discussion

Using the KARMA cohort, including 570 patients with breast cancer and 60,237 healthy control subjects, we generated a comparatively simple breast cancer risk prediction model for clinical use. Exploiting three fully automatically measured mammographic features enabled identification of women at an approximately ninefold greater risk of developing breast cancer when we compared the high- and low-risk groups (Table 4). In the full model, taking HRT use, family history of breast cancer, and menopausal status into consideration, the AUC reached 0.71 (Table 5).

Several studies have shown mammographic density to be an excellent predictor of breast cancer risk where women with high breast density have a four to six times higher risk than women with low breast density [16]. Reassuringly, we observed a relative risk of 4.8 (95% CI 2.6–8.8) when we compared the highest with the lowest cBIRADS scores (Table 3). Comparing the highest with the lowest numbers of microcalcifications and masses each gave significant relative risks of approximately 2 (Table 3). In addition, the difference in number of microcalcifications between the breasts gave a risk of 2.8 (95% CI 1.8–4.5).

It should be underlined that our model identifies women at short-term risk of being diagnosed with breast cancer. These women are in their later progression but earlier stage, have a negative screening mammogram result, and are within 2 years of being diagnosed with either an interval cancer or a cancer at the next screening visit. The interval cancers were also shown to be at the highest risk (Additional file 1: Table S2). There are studies presenting extremely high relative risks of mammographic density (OR 17.8) for interval cancer [17]. We would have got similar results if we had not considered that interval cancers should be compared with control subjects also having clinical examinations and not with control subjects having an ordinary scheduled screening mammogram.

Adding the clinical observation that differences in density, microcalcifications, and masses between the breasts are indicators of malignancy developed our model further. It has long been known that breast asymmetry is a risk factor for breast cancer [18]. In our model, the influence on risk from breast asymmetry was as strong as that from the total number of microcalcifications and masses (Table 3). This means that the risk association with the total number of microcalcifications was driven mainly by the increase of microcalcifications in one of the breasts. This indicates that the difference in calcifications between the breasts was the important risk marker for malignancy, although a dose-response relationship with the total number of microcalcifications might be seen with multifocal tumors. The risk from breast asymmetry was also significantly higher in interval cancers than in screening-detected cancers (Additional file 1: Table S2).

The biology behind microcalcifications is not well understood. One hypothesis is that epithelial cells acquire mesenchymal characteristics and, as a sign of carcinogenic transformation, become capable of producing breast microcalcifications [19]. Because we found microcalcifications to be more abundant on the tumor side and that density was almost doubled in cases versus control subjects, it could be argued that microcalcifications are signs of a precursor lesion, whereas density is a general sign of increased breast cancer risk. In our full model including mammographic density, microcalcifications, and masses, the AUC reached 0.71, as compared with the Tyrer-Cuzick and Gail models, with AUCs 0.63 and 0.56, respectively (Table 5) [1, 20]. We thus found that our model added substantial discriminatory effect. More than half (*N* = 284) of the total number of patients with breast cancer (*n* = 570) who developed breast cancer during the study follow-up were predicted at baseline in the highest quintile of the 2-year absolute risk score (Additional file 1: Table S4). It should also be noted that the relative risks of cancer in situ were significantly higher than invasive cancer when we compared women with and without microcalcifications (Additional file 1: Table S2), although the model predominantly identified increased number of invasive cancers at higher risk levels compared with the Tyrer-Cuzick and Gail models (Additional file 1: Table S4). All models showed the same tendency with increased numbers of invasive and in situ cases by increased levels of risk.

In Sweden, approximately 6 of 1000 women are diagnosed with breast cancer at each round of biannual screening [21]. We managed to identify a low-risk group of approximately 10% of all women in which 1 woman in 1000 will be diagnosed with breast cancer. In contrast, in the highest risk category, 20 of 1000 women will have cancer detected within 2 years (Fig. 1).

The individualized screening protocol requires information on mammographic features, age, BMI, family history of breast cancer, use of HRT, and menopausal status of the woman. The mammographic measures are fully automated and require approximately 120 seconds of computation time to be generated. The remaining risk factors are easily collected through an online questionnaire at the time of the mammography visit. External validations of the result are needed to verify the performance of our risk model. It will be of utmost importance to understand which types of tumors the model predicts. Most established models target receptor-positive and highly differentiated tumors (i.e., tumors seen as less aggressive). In future studies, it will also be of importance to understand the relationship between the localization of the mammographic features and subsequent tumors and how different cut-off points for defining interval cancer will ﻿influence the risk estimates.

The KARMA cohort is large, but the follow-up time is just some few years. The obvious weakness of our study is the low number of breast cancer cases. For women with missing data on a risk factor, imputation was performed according to the protocol established with each risk model. We calculated a cBIRADS score that mimics the established BI-RADS score to help clinical implementation, but we do not know how the true BI-RADS score would influence our model. As a unique strength, we built our model on one of the few existing population-based prospective screening cohorts with detailed information on factors that possibly influence the risk of breast cancer.

## Conclusions

Our model includes three mammographic features that could easily be derived from raw mammograms. By adding information on some few established risk factors, it has the potential to individualize screening and improve clinical care by identifying women in need of additional examination procedures. At the same time, there may be a substantial proportion of women who will have very little benefit from mammography screening, owing to their low risk of breast cancer.

## Additional file

Additional file 1: Table S1. Relative risk of developing breast cancer in relation to mammographic density, number microcalcifications and number masses. **Table S2.** Relative risks on developing breast cancer in relation to tumor invasiveness and mode of detection. **Table S3.** Final model including main effects of risk factors, beta coefficients, standard errors and p-values. **Table S4.** Number of breast cancer cases diagnosed during study follow-up stratified by predicted risks at baseline in the Karma cohort. Supplementary Method 1. Supplementary Method 2. Supplementary Method 3. (DOCX 59 kb)

## Abbreviations

BI-RADS: Breast Imaging Reporting and Data System
BMI: Body mass index
CAD: Computer-aided detection
cBIRADS: Computer-generated Breast Imaging Reporting and Data System score
CC: Craniocaudal
HRT: Hormone replacement therapy
iCARE: Individualized Coherent Absolute Risk Estimator
KARMA: Karolinska Mammography Project for Risk Prediction of Breast Cancer
LR: Likelihood ratio
MLO: Mediolateral oblique
