# Supplementary material for: A clinical model for identifying the short-term risk of breast cancer
Source: Breast Cancer Res. 2017 Mar 14;19:29. doi: 10.1186/s13058-017-0820-y (PMC5348894; doi:10.1186/s13058-017-0820-y)
Supplement: Additional file 1: Table S1. — Relative risk of developing breast cancer in relation to mammographic density, number microcalcifications and number masses. Table S2. Relative risks on developing breast cancer in relation to tumor invasiveness and mode of detection. Table S3. Final model including main effects of risk factors, beta coefficients, standard errors and p-values. Table S4. Number of breast cancer cases diagnosed during study follow-up stratified by predicted risks at baseline in the Karma cohort. Supplementary Method 1. Supplementary Method 2. Supplementary Method 3. (DOCX 59 kb) [file 13058_2017_820_MOESM1_ESM.docx]

**Table S1. Relative risk of developing breast cancer in relation to mammographic density, number microcalcifications and number masses**

| **Mammographic features** | **No of cases** | **No of controls** | **HR^1^ (95% CI)** | **HR^2^ (95% CI)** |
| --- | --- | --- | --- | --- |
| Percent mammographic density (cBIRADS 1 as reference) | 26 | 193 | 1.0 | 1.0 |
| 2 | 174 | 829 | 2.1 (1.4-3.2) | 2.1 (1.3-3.3) |
| 3 | 189 | 593 | 3.6 (2.3-5.6) | 3.3 (2.0-5.4) |
| 4 | 44 | 117 | 4.9 (2.8-8.6) | 4.8 (2.6-8.8) |
|  |  |  |  |  |
| Number of microcalcification (0 microcalcifications as reference)^3^ | 272 | 1401 | 1.0 | 1.0 |
| 1 | 59 | 147 | 1.9 (1.4-2.5) | 1.9 (1.4-2.6) |
| 2 | 49 | 81 | 2.4 (1.7-3.3) | 2.4 (1.7-3.4) |
| 3 | 31 | 53 | 2.4 (1.7-3.4) | 2.4 (1.6-3.5) |
| 4 | 22 | 50 | 2.0 (1.3-3.1) | 2.0 (1.3-3.2) |
| Number of masses (0 masses as reference) | 136 | 655 | 1.0 | 1.0 |
| 1 | 193 | 724 | 1.2 (1.0-1.5) | 1.1 (0.9-1.4) |
| 2 | 72 | 274 | 1.2 (0.9-1.6) | 1.1 (0.8-1.5) |
| 3 | 25 | 62 | 1.6 (1.0-2.5) | 1.6 (1.0-2.6) |
| 4 or more | 7 | 17 | 1.7 (0.8-3.5) | 1.7 (0.8-3.6) |

^1^HR (hazard ratios) adjusted for age, BMI, CI = confidence interval.

^2^HR (hazard ratios) adjusted for age, BMI, mammographic density, microcalcifications, masses, breast cancer in family, menopausal status and current use of HRT.

^3^Categoy 0 means 0 calcifications, 1 is 1-10 calcifications. The corresponding number for 2, 3, 4 is 11-20, 21-40, >40 calcifications respectively.

**Table S2. Relative risks on developing breast cancer in relation to tumor invasiveness and mode of detection**

|  | **Invasive BC (N=383)** | **In-situ BC (N=50)** | **Difference by invasiveness** | **Screening BC (N=275)** | **Interval BC (N=158)** | **Difference by detection** |
| --- | --- | --- | --- | --- | --- | --- |
| **Study participant and mammographic risk factors** | **HR^1^ (CI)** | **HR^1^ (CI)** | **p-value^2^** | **HR^1^ (CI)** | **HR^1^ (CI)** | **p-value^2^** |
| Current use of HRT | 1.3 (0.8-2.0) | 2.1 (0.9-4.9) | 0.16 | 1.6 (1.0-2.6) | 1.0 (0.5-2.2) | 0.78 |
| Breast cancer in family | 1.3 (1.0-1.7) | 1.7 (0.8-3.3) | 0.48 | 1.4 (1.0-1.9) | 1.1 (0.7-1.7) | 0.20 |
| Mammographic density (per cBIRADS) | 1.8 (1.5-2.1) | 1.9 (1.1-3.0) | 0.54 | 1.5 (1.3-1.9) | 2.1 (1.6-2.7) | 3.4x10^-15^ |
| Microcalcifications category 1 or higher vs. no calcifications | 2.0 (1.6-2.5) | 4.0 (2.2-7.4) | 0.03 | 1.6 (1.2-2.1) | 2.6 (1.8-3.7) | 5.8x10^-8^ |
| Masses vs. no masses in breast | 1.2 (1.0-1.5) | 1.2 (0.7-2.1) | 0.69 | 1.4 (1.1-1.8) | 1.1 (0.8-1.5) | 9.8x10^-8^ |
|  |  |  |  |  |  |  |
| *Difference between left and right breast^3^* |  |  |  |  |  |  |
| Percent mammographic density | 3.9 (2.5-6.1) | 1.3 (0.4-3.7) | 0.16 | 1.6 (0.9-2.7) | 3.9 (1.6-9.0) | 4.1x10^-7^ |
| Number of micro-calcifications | 2.2 (1.7-2.9) | 6.3 (3.0-13.2) | 0.01 | 2.0 (1.1-3.7) | 5.3 (2.6-10.7) | 2.5x10^-8^ |
| Number of masses | 1.5 (0.9-2.4) | 1.3 (0.4-4.8) | 0.62 | 0.9 (0.5-1.9) | 1.6 (0.6-4.4) | 8.5x10^-9^ |

^1^Hazard Ratio HR and Wald 95% confidence intervals CI from conditional logistic regression model calculating average relative risks over the follow-up period. HR adjusted for age, BMI, mammographic density, microcalcifications, masses, use of hormonal replacement therapy, mother and/or sister diagnosed with breast cancer. Controls matched to cases on timing of examination procedure; screening detected cases are compared to controls with screening mammograms and non-screen detected cases in clinical routine to controls with clinical examinations.

^2^Difference was estimated as the significance of the interaction term between the risk factor and invasiveness, and detection mode, respectively.

^3^Difference between left and right breast is a reciprocal number in range 0 – 1 and defined as one divided by the standard deviation of left and right breast measures for each woman.

**Table S3. Final model including main effects of risk factors, beta coefficients, standard errors and p-values**

| **Predictor** | **Beta coefficient** | **Standard error** | **p-value** |
| --- | --- | --- | --- |
| *Pre-menopausal women* |  |  |  |
| BMI | -0.02844 | 0.02247 | 0.21 |
| Current use of HRT | 0.32873 | 0.43248 | 0.45 |
| Breast cancer in family | 0.42086 | 0.20508 | 0.04 |
| Percent mammographic density | 0.68075 | 0.14681 | 2.8x10^-6^ |
| Mammographic density, absolute difference between breasts^1^ | -1.02888 | 0.41060 | 1.2x10^-2^ |
| Microcalcifications, absolute difference between breasts^1^ | -1.15361 | 0.21907 | 1.4x10^-7^ |
| Interaction: percent mammographic density * masses | 0.09739 | 0.04305 | 0.1198 |
|  |  |  |  |
| *Post-menopausal women* |  |  |  |
| BMI | 0.03712 | 0.01278 | 0.004 |
| Current use of HRT | 0.36087 | 0.23861 | 0.13 |
| Breast cancer in family | 0.40149 | 0.16763 | 0.02 |
| Percent mammographic density | 0.49514 | 0.10564 | 3.0x10^-6^ |
| Mammographic density, absolute difference^1^ | -1.15534 | 0.28124 | 4.0x10^-5^ |
| Microcalcifications, absolute difference^1^ | -0.77451 | 0.15571 | 6.6x10^-7^ |
| Interaction: percent mammographic density * masses | 0.08732 | 0.03170 | 5.9x10^-3^ |

^1^The absolute difference measure was entered into the model as a reciprocal transformed predictor.

**Table S4. Number of breast cancer cases diagnosed during study follow-up stratified by predicted risks at baseline in the Karma cohort**

|  | **Quintile of predicted 2-year absolute risk** | | | | | **Beta** | |
| --- | --- | --- | --- | --- | --- | --- | --- |
| **Diagnosed cases, models** | Q1 | Q2 | Q3 | Q4 | Q5 | per Q^1^ | p-value^2^ |
| *Invasive cases (N)* |  |  |  |  |  |  |  |
| Current model | 27 | 58 | 61 | 108 | 217 | 42.8 | 0.03 |
| Tyrer-Cuzick | 40 | 67 | 97 | 107 | 160 | 27.8 | 0.01 |
| Gail | 90 | 71 | 83 | 89 | 138 | 11.2 | 0.19 |
|  |  |  |  |  |  |  |  |
| *In-situ cases (N)* |  |  |  |  |  |  |  |
| Current model | 4 | 4 | 7 | 17 | 67 | 13.9 | 0.09 |
| Tyrer-Cuzick | 5 | 17 | 16 | 20 | 41 | 7.5 | 0.04 |
| Gail | 13 | 17 | 19 | 18 | 32 | 3.9 | 0.06 |
|  |  |  |  |  |  |  |  |
| *All cases (N=570)* |  |  |  |  |  |  |  |
| Current model | 31 | 62 | 68 | 125 | 284 | 56.7 | 0.04 |
| Tyrer-Cuzick | 45 | 84 | 113 | 127 | 201 | 35.3 | 0.01 |
| Gail | 103 | 88 | 102 | 107 | 170 | 15.1 | 0.14 |

Quintiles of 2-year absolute risks were calculated at baseline in the full Karma cohort for each model. The number of breast cancer cases diagnosed during the study follow-up time was tabulated into their predicted baseline risk quintile.

^1^Average increase in number of breast cancer cases identified during follow-up per quintile of absolute 2-year risks predicted at baseline. Average increase was estimated with linear regression.

^2^p-value in linear regression tested for beta-coefficient = 0.

**Supplementary Method 1**

Stratus creates density estimates on digital processed mammograms in a training session based on four thousand mammograms per type of mammography machine. These density estimates are then validated in an independent and equal sized set of processed mammograms. The final use of Stratus is to measure density on digital processed mammograms available in big resources worldwide.

The training session estimates density on the processed copy of the same mammogram where a raw copy also is available. The raw copy is used as a standard for measuring density with the reference method. The fully automated area based measurement from iCAD (iReveal®, iCAD, Nashua, NH, USA), approved by the US Food and Drug Administration, is used as the reference measure. iReveal is an area based method that complies with the American College of Radiology recommendations [^[[1]](#endnote-1)^]. iReveal divides breast composition into four BI-RADS categories, A) almost entirely fatty, B) scattered areas of fibro-glandular density, C) heterogeneously dense, and D) extremely dense.

The image analysis in Stratus is based on the ImageJ programming framework developed by National Institutes of Health [^[[2]](#endnote-2)^] and runs as a Java based program within this framework. The breast area is marked using the Intermodes threshold method. Features are further identified in a cycle of multiple threshold methods and measures multiple features in each cycle. Mammogram acquisition parameters are also added to the measures. All measures are compiled into one row with more than one thousand variables per mammogram.

In the second step Stratus learns to estimate the reference density measure from the one thousand image features using machine learning technique. In greater detail, the breast area and dense area from the reference measure are transformed to approximate the normal distribution. Scaled principal components are further performed on the image features. These are then entered as input parameters in a penalized linear regression. The penalized linear regression calculated Lambda-fits in multiple loops using the generalized lasso regression model with folded cross-validation. The mean lambda is calculated and used for the final model fit. The estimated dense area and breast area measures are then back transformed to original density distribution. This builds up the Stratus estimates per vendor and model of mammography machine. These density estimates are then applied to new datasets of digital processed images. The processed image mammogram type is the most commonly available worldwide.

**Supplementary Method 2**

Stratus measures percent mammographic density on a continuous scale between 0 and 100 percent. Stratus also use cut-points on the continuous scale (2%, 18%, 49%) to be calibrated with the clinically accepted BI-RADS breast composition categorization. We used the full Karma cohort to tabulate the percentages of women in the Stratus cBIRADS categories; and to compare them with the corresponding percentages and categorizations of the mammographic density measurement software iReveal [^[[3]](#endnote-3)^], Philips [^[[4]](#endnote-4)^], and Volpara [^[[5]](#endnote-5)^]. All software used cut-points on their continuous scale. As can be seen from the Table below Stratus, iReveal and Philips ended up with more or less the same proportion of women in the different categories. Volpara has a slightly different proportion of women in the first two categories. Non-significant differences between expected and observed percentages were found for Stratus, iReveal, and Philips, (chi-square test p=0.97, p=0.99, 0=0.91), and for Volpara, p=0.20.

|  | **Density measurement software** | | | |
| --- | --- | --- | --- | --- |
| **Approximated BI-RADS categories** | **Stratus** | **iReveal** | **Philips** | **Volpara** |
|  |  |  |  |  |
| 1. (almost entirely fatty breasts), % | 12 | 11 | 12 | 19 |
| 2. (scattered areas of fibro-glandular tissue), % | 40 | 41 | 43 | 30 |
| 3. (heterogeneously dense), % | 38 | 38 | 36 | 38 |
| 4. (extremely dense), % | 10 | 10 | 9 | 13 |
| Total % women | 100 | 100 | 100 | 100 |

**Supplementary Method 3**

Karma recruited 70,877 women from January 2011 to March 2013. 570 incident breast cancer cases were identified through register linkage in October 2015. 137 women did not have complete questionnaire information on all risk factors used in the MammoDetect risk score. 120 women did not report use of HRT and 11 women did not report BMI. 6 women did neither report use of HRT or BMI. The remaining 433 women and 1,732 age matched controls were used for estimating the relative risks for the MammoDetect model.

Absolute risks were calculated for the full Karma cohort with 570 incident breast cancer cases and the 60,237 controls which remained after 3,126 prevalent breast cancer cases and 7,514 controls with lacking mammograms were excluded.

Imputation was done prior to calculating absolute risks for women with a missing risk factor. The iCARE method defined a single model which was used to derive estimates for all risk factors for all women [^[[6]](#endnote-6)^]. iCARE defined a risk score R for each woman i, R_i_, based on a set of risk factors Z_i_ for that woman (age, BMI, mammographic density, microcalcifications, masses, use of HRT, menopause status, and family history of breast cancer). The risk score was defined as a linear predictor β^T^Z_i_ of the beta-coefficients β in Z, i.e. the log relative risk model. The risk score R_i_ was partitioned into $R\begin{matrix} o \\ iP \end{matrix}$ + $R\begin{matrix} u \\ iP \end{matrix}$, where P indexed the observed pattern of missing risk factors, and $R\begin{matrix} o \\ iP \end{matrix}$ denoted the corresponding observable (o) components of the risk score (defined as β$\begin{matrix} oT \\ p \end{matrix}Z\begin{matrix} o \\ iP \end{matrix}$), and $R\begin{matrix} u \\ iP \end{matrix}$ denoted the unobservable (u) components of the risk score (defined as β$\begin{matrix} uT \\ p \end{matrix}Z\begin{matrix} u \\ iP \end{matrix}$). The partitioning was defined based on what columns in the original model design matrix could be specified by the observed set of risk factors for each risk factor level and for each woman. The absolute risk R_i_ could then be derived by averaging the possible values for the unobserved component of the risk score based on the values from the observed components of the risk score. Non-parametrical estimation of the conditional distributions pr($r\begin{matrix} m \\ iP \end{matrix}$ | $R\begin{matrix} o \\ iP \end{matrix}$) was done using the Karma cohort risk factor distributions. This means that absolute risk was calculated for each woman with missing risk factors by identifying women in the full Karma cohort with similar observable risk factors $R\begin{matrix} o \\ iP \end{matrix}$. The risk for the women with missing risk factors was estimated as the average risk AR($R\begin{matrix} o \\ p \end{matrix}$, $r\begin{matrix} u \\ p \end{matrix}$) found for the women with complete risk factors. The observable risk scores $R\begin{matrix} o \\ iP \end{matrix}$ were generalized into categories of percentile strata, and the risks for women with missing risk factor data was estimated as the average of all AR($R\begin{matrix} o \\ p \end{matrix}$, $r\begin{matrix} u \\ p \end{matrix}$) in the matching stratum.

1. . American College of Radiology. Breast Imaging Reporting and Data System (BI-RADS) 5. Reston, Va : s.n., 2013. [↑](#endnote-ref-1)
2. . Schneider, C.A., Rasband, W.S., Eliceiri, K.W. "NIH Image to ImageJ: 25 years of image analysis". Nature Methods 9, 671-675, 2012 [↑](#endnote-ref-2)
3. . <http://www.icadmed.com>. Accessed 1 December 2016. [↑](#endnote-ref-3)
4. . Y. Machida, M. Tozaki, T. Yoshida, A. Saita, M. Yakabe, K. Nii, "Feasibility study of a breast density measurement within a direct photon-counting mammography scanner system," Jpn. J. Radiol. 32 (2014). [↑](#endnote-ref-4)
5. . Lee et al.; Comparison of mammographic density estimation by Volpara software with radiologists' visual assessment: analysis of clinical-radiologic factors affecting discrepancy between them; Acta Radiol. 2015 Sep;56(9):1061-8. doi: 10.1177/0284185114554674. Epub 2014 Oct 22. [↑](#endnote-ref-5)
6. . Maas P, Barrdahl M Joshi AD et al; Breast Cancer Risk From Modifiable and Nonmodifiable Risk Factors Among White Women in the United States; JAMA Oncol. 2016 May 26. doi: 10.1001/jamaoncol.2016.1025; http://dceg.cancer.gov/tools/analysis/icare. Accessed 28 December 2016. [↑](#endnote-ref-6)
